# Raman Computational and Experimental Studies of Dopamine Detection

**DOI:** 10.3390/bios7040043

**Published:** 2017-09-28

**Authors:** John D. Ciubuc, Kevin E. Bennet, Chao Qiu, Matthew Alonzo, William G. Durrer, Felicia S. Manciu

**Affiliations:** 1 Department of Physics, University of Texas at El Paso, El Paso, TX 79968, USA; jdciubuc@miners.utep.edu (J.D.C.); chaoqiu66@gmail.com (C.Q.); malonzo6@miners.utep.edu (M.A.); wdurrer.utep.edu (W.G.D.); 2 Department of Biomedical Engineering, University of Texas at El Paso, El Paso, TX 79968, USA; 3 Division of Engineering, Department of Neurologic Surgery, Mayo Clinic, Rochester, MN 55905, USA; Bennet.Kevin@mayo.edu; 4 Border Biomedical Research Center, University of Texas at El Paso, El Paso, TX 79968, USA

**Keywords:** dopamine detection, label-free, surface-enhanced Raman spectroscopy, physiological levels, biosensors, computer simulation, silver nanocolloids

## Abstract

A combined theoretical and experimental analysis of dopamine (DA) is presented in this work with the objective of achieving more accurate detection and monitoring of this neurotransmitter at very low concentrations, specific to physiological levels. Surface-enhanced Raman spectroscopy on silver nanoparticles was employed for recording DA concentrations as low as 10^−11^ molar. Quantum chemical density functional calculations were carried out using Gaussian-09 analytical suite software. Relatively good agreement between the simulated and experimentally determined results indicates the presence of different DA molecular forms, such as uncharged DA^±^, anionic DA^−^, and dopaminequinone. Disappearance of the strongest bands of dopamine around 750 cm^−1^ and 790 cm^−1^, which suggests its adsorption onto the metallic surface, is not only consistent with all of these DA configurations, but also provides additional information about the analyte’s redox process and voltammetric detection. On the other hand, occurrence of the abovementioned Raman lines could indicate the formation of multilayers of DA or its presence in a cationic DA^+^ form. Thus, through coordinated experiment and theory, valuable insights into changes observed in the vibrational signatures of this important neurotransmitter can be achieved for a better understanding of its detection at physiological levels, which is crucial if further optovoltammetric medical device development is envisioned.

## 1. Introduction

Surface-enhanced Raman spectroscopy (SERS) was discovered over forty years ago through the enhancement of the Raman signal that was observed for pyridine adsorption on soft silver electrodes [1]. Since then, SERS has gone through multiple advances in understanding and expanding the technique. The SERS enhancement factor is now known to be due to two primary phenomena: electromagnetic (EM) and chemical (CM) enhancement mechanisms [2]. Electromagnetic enhancement serves as the bulk of the enhancement factor due to local EM field effects, providing enhancement to the order of 10^4^–10^10^ [2,3]. Chemical enhancement is due to metal-adsorbate charge resonance and interactions, and has a lower magnitude influence, contributing a factor of 10^2^–10^3^ [2,3,4]. The level of enhancement that can be achieved in SERS depends, of course, on the nature of the metallic nanoparticles themselves. Furthermore, adsorbates at specific locations in the proximity of the nanoparticles exhibit Raman ‘hot-spot’ effects; such locations are SERS-active sites, at which an impressive gain in the amplification of the Raman signal is observed [2,5,6]. SERS has even led to the study of single molecules, achieving an enhancement factor of over 10^10^ through the adhesion of nanoparticles to individual molecules [5,6,7,8].

Dopamine (DA) is the focus of the SERS study presented in this paper. It is one of the most studied neurotransmitters, as it is centrally involved in many neurological mechanisms, ranging from those of the extrapyramidal motor tracts of the central nervous system to that of psychostimulant addiction [9,10,11,12,13]. As such, in addition to the relevance of detecting it accurately, it has also been used as a potent drug for activating receptors in the brain, heart muscle, kidney, and gut [9,10,11,12,13]. The level of receptor activation strongly depends on the dose administrated. The need for its accurate detection at physiological levels is largely the reason for the development of various techniques, ranging from electrochemistry [14], chromatography [15], and gravimetry [16,17], to optical spectroscopy [5,6,7,8]. From a chemical perspective, its ionizable functional groups—an ethyl ammonium, which acts as a proton acceptor, and two phenolic hydroxyls, which act as donors—can participate in DA molecular bonding. At physiological levels, the dominant presence of ionized DA has been reported from both experimental [14,15,18] and theoretical [16,17,19] results.

Besides the degree of adsorption and polarizability of a particular analyte, another factor that is relevant to our work here, because it can significantly influence the SERS signal, is analyte orientation in the vicinity of the metallic nanoparticles [5,6,8,19]. Under its influence, SERS data exhibit unique characteristics, commonly showing rapid changes in vibrational intensities. In addition, analyte-nanoparticle orientation with respect to the Raman excitation source can definitely affect the outcome of such a measurement. As a result, in some cases, the relative Raman intensities of some vibrational lines can be completely suppressed. For example, there is no observable activity of DA vibrations at 750 and 795 cm^−1^, vibrations associated with either the in-plane phenolic ring bending mode or with the out-of-plane O–H and C–H bending modes [19,20]. On the other hand, if DA orientation is optimum, remarkable increases in Raman signatures are obtained. Usually, vibrations associated with larger components of polarizability in the direction normal to the silver nanoparticle surface are enhanced more.

The aid of computational methods and powerful computers currently offers considerable advantages in identifying structural modifications of analytes and their consequent influence on vibrational outcomes. There is a general consensus that density functional theory (DFT) provides sufficiently consistent results that are in relatively good agreement with experimentally determined molecular frequencies. Thus, prediction of DA orientations and structural modifications through computational analysis allows for an effective understanding of the resulting Raman spectrum.

Although this study mainly aims to make valuable predictions of Raman intensities and wavenumber locations through combinatorial computational and experimental analysis, such evaluations are of importance for understanding other physicochemical phenomena resulting from currently employed fast-scan cyclic voltammetric devices. In this context, it is worth mentioning that roughened electrode surfaces have previously been used as SERS substrates [1,21,22]. Moreover, the current Raman evaluations are, in fact, based on experimental results of dopamine recordings for as low as 10^−11^ molar. The ultrasensitivity of the experimental data, which are definitely at the normal physiological levels of DA, is another indication of the value of the results discussed in this work.

## 2. Materials and Methods

### 2.1. Materials and Equipment

Silver nitrate (AgNO_3_, >99%), sodium borohydride (NaBH_4_, >99%), and citric acid trisodium salt dihydrate (C_6_H_5_Na_3_O_7_·2H_2_O, 99%) reagents purchased from Sigma-Aldrich and ACROS, respectively, were used for the synthesis of silver nanoparticles (Ag NPs) following a previously reported procedure [23]. Briefly, the chemical process consists of mixing 20 mL of 1% (*w*/*v*) citrate solution with 75 mL of ultrapure water and heating the solution to 80 °C until stable. Next, 1.7 mL of 1% (*w*/*v*) AgNO_3_ and 2 mL of 0.1% (*w*/*v*) freshly prepared NaBH_4_ solutions were added to the mixture. The reaction solution was kept at 80 °C under vigorous stirring for 30 min and cooled to room temperature. Purification of the resulting solution to remove the excess of organic and unreacted impurities was then performed several times; it consisted of washing with ultrapure water and centrifugation. Next, 90 μL of synthesized Ag NPs was mixed with 10 μL a 10^−10^ molar solution of DA. The mixed solutions were sonicated for 20 s, then drop-cast on clean cover slips to form dense, uniform Ag NP thin films.

The Raman measurements were acquired at ambient conditions in a backscattering geometry with an *alpha 300RAS* WITec system (WITec GmbH, Ulm, Germany). A 532-nm excitation of a frequency-doubled neodymium-doped yttrium–aluminum–garnet (Nd:YAG) laser that was restricted to a power output of about 100 μW to avoid sample damage and a 20× objective (Olympus, Tokyo, Japan) were used. Time series Raman spectra, each of 200 milliseconds, were recorded. WITec Control 1.60 software was employed for fast data acquisition.

### 2.2. Computational Analysis

The quantum chemical density functional calculations were carried out using Gaussian-09 analytical suite software. The DA molecule was first energetically optimized for all of its forms, neutral (DA^0^), anionic (DA^−^), and cationic (DA^+^), and their vibrational frequencies were computed using the Becke three hybrid exchange [24] and Lee-Yang-Parr correlation functional [25], B3LYP. A Pople split valence diffused and polarized 6-311++G(d,p) basis set was used for such calculations. The Raman activities, *S_i_*, obtained from the Gaussian-09 software were further converted into relative Raman intensities (*I_i_*) using the following equation [26,27,28]:

(1) $$I_{i}=\frac{f\;S_{i}\;{\left(\nu_{0}-\nu_{i}\right)}^{4}}{\nu_{i}\left[1-\exp\left(-\frac{hc\nu_{i}}{kT}\right)\right]}$$

where *ν*_0_ is the excitation frequency (532 nm = 18,796.99 cm^−1^), *ν_i_* is the vibrational wavenumber of the *i^th^* normal mode, *h* is the Planck’s constant, *c* is the speed of light, and *k* is the Boltzmann constant. A normalization factor *f* = 1e^−12^ was used for all peak intensities and a value of 293.15 Kelvin was used for the temperature, *T*. Lorentzian band shapes were also applied to the resulting Raman intensity spectrum, using a full width at half maximum (FWHM) of 7 cm^−1^. To enable data plotting, parsing of the Gaussian-09 Raman output data, which was completed through an in-house algorithm developed in the C++ language utilizing the Qt framework, as well as conversion through MATLAB version r2016a, was performed. 

## 3. Results

Group theory predicts that both planar and non-planar species are expected in all of the DA forms (i.e., DA^0^, DA^−^, and DA^+^), which all have C_S_ point group symmetry [19,26]. Furthermore, both species are Raman and infrared (IR) active. For Raman in particular, the polarized bands are mainly associated with in-plane vibrations and the depolarized ones with out-of-plane vibrations.

**Figure 1:**
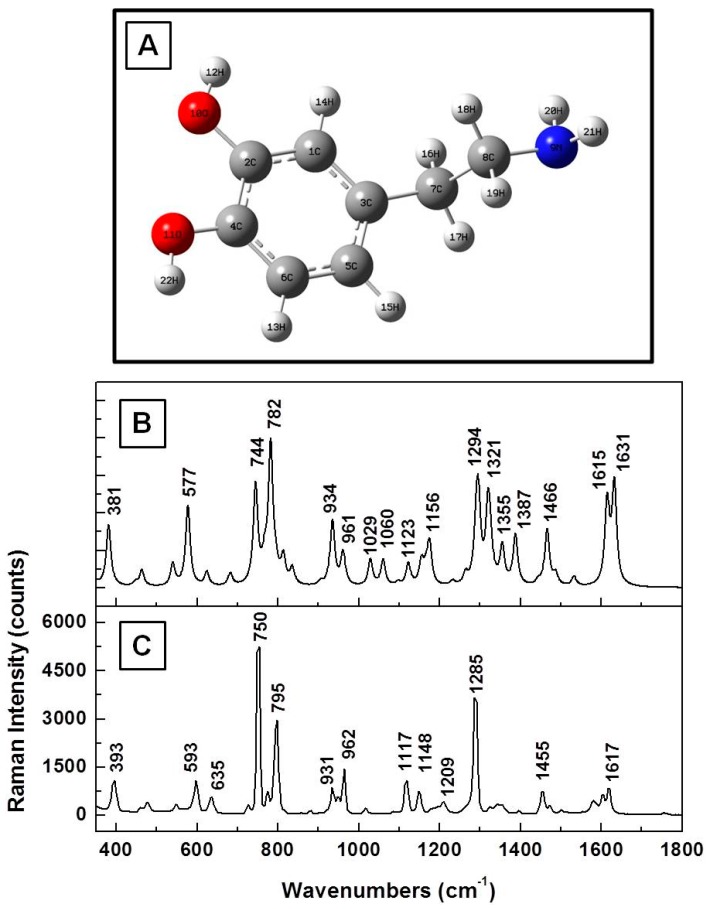
(**A**) Dopamine structural representation in neutral state, DA^0^. Red and blue colors were used for oxygen and nitrogen atoms, respectively; (**B**,**C**) Theoretically calculated and experimentally measured Raman vibrations of dopamine, respectively. The Raman spectrum was recorded for the standard dopamine powder.

The optimized molecular structure of DA^0^ and the calculated Raman vibrations using the Gaussian software are presented in Figure 1A,B, respectively. We also show in Figure 1C the experimental Raman spectrum of solid dopamine (i.e., of DA powder), for easier comparison between measurements and simulations. There is relatively good agreement between the peaks of the theoretically estimated frequencies, which have been scaled by a factor of 0.98, and the observed vibrations, except for some variations in the peak intensities. Typically, scaling factors have been used in the literature to overcome systematic empirical errors originating from the force field constants employed in quantum mechanical approaches [19,26]. Although it is a debated subject [29], as a single uniform scaling factor (or frequency scaling) cannot entirely account for all errors, we still consider it in this work, as our perspective stems from a desire to obtain a reliable understanding of vibrational assignments as compared with experimentally observed phenomena. Even higher level theoretical calculations often cannot perfectly account for the complexity of experimental specifics in their entirety.

Although similar calculated frequencies for DA have been reported in the literature [19], some repetition of results in research is inevitable and contributes to such supportive purposes as cross-checking and confirmation of reproducibility. At a glance, there are discrepancies of 10 ± 2 cm^−1^, on average, between the experimentally determined and estimated values. For the unscaled frequencies (data not reported here), these discrepancies become larger at higher frequency, indeed suggesting an overestimation of the force field constant used in Gaussian simulations. The measured and computationally estimated values of the main vibrational modes along with their assignments are summarized in Table 1. The calculated values presented include a scaling factor of 0.98. The assignments were made by direct visualization with Gaussview software, as well as by comparison with the literature [28,29,30,31].

**Figure 2:**
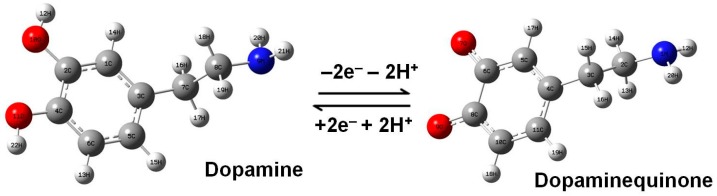
The redox process of dopamine consisting of the transfer of two electrons and two protons. The structural representations of neutral dopamine and dopaminequinone are also presented and appropriately labeled.

Based on previous studies, the deprotonation of dopamine is expected when it is dissolved in water, which can result in the molecular transformation of DA into dopaminequinone, besides its other forms mentioned above (e.g., DA^−^, and DA^+^) [19,32,33,34]. Since this process is likely to occur in the preparation of our 10^−11^ M dopamine solution, we schematically present it in Figure 2, together with molecular representations of the two compounds. The quinonic form is considered first due to the obvious symmetry of the DA molecule as regards the O–H and N–H bonds, which is known to result in a one-step redox reaction involving two electrons and two protons. The anionic and cationic forms are also investigated in this work.

**Figure 3:**
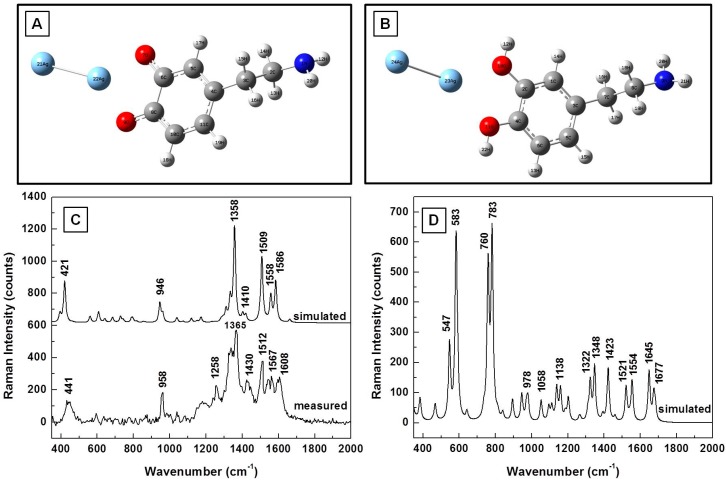
(**A**,**B**) Dopaminequinone and neutral DA^0^ structural representations in the proximity of a silver dimer after energy optimization, respectively; (**C**) Theoretically estimated and experimentally recorded Raman vibrational spectra of dopaminequinone; (**D**) Theoretically calculated Raman spectrum of dopamine in the proximity of silver.

The effect of a SERS environment on the Raman vibrational frequencies of dopaminequinone and neutral DA^0^ is further analyzed from theoretical and experimental perspectives in Figure 3A–D. The structural representations of the analyte in the proximity of silver after energy optimization using an LanL2DZ basic set that takes into consideration the pseudopotentials for metal atoms are shown in Figure 3A,B. A comparison of the estimated Raman frequencies of dopaminequinone and the Raman results that were obtained experimentally, which are presented in Figure 3C, not only shows good agreement, but, more importantly, confirms the disappearance of dominant DA Raman vibrations at 750 cm^−1^ and 795 cm^−1^, in both the simulation and the experimental data. However, this disappearance does not occur for the calculated vibrational lines of neutral DA^0^ in the proximity of Ag NPs, as observed in Figure 3D. On the contrary, they are the most intense Raman features seen in this figure, besides two other vibrations around 550 cm^−1^ and 580 cm^−1^. The stronger interaction of the dopaminequinonic form with silver could be attributed to a somewhat greater electronegativity of oxygen atoms in this configuration than in DA^0^. Dominant Raman vibrations around 1360 cm^−1^ and 1510 cm^−1^ are observed in Figure 3C for dopaminequinone interaction with Ag NPs.

**Figure 4:**
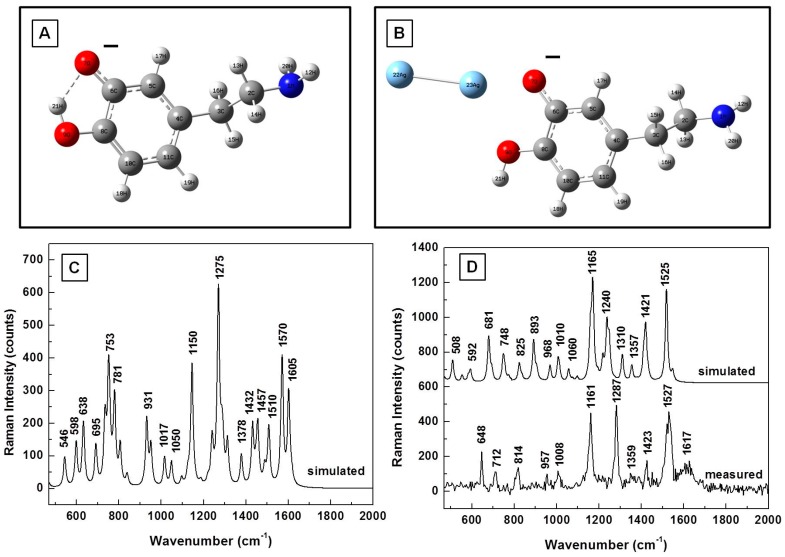
(**A**,**B**) Structural representations of the anionic form of dopamine without and with the silver dimer, respectively; (**C**) Theoretically calculated Raman vibrations of DA^−^; and (**D**) Theoretically calculated and experimentally measured Raman vibrations of the anionic form in the proximity of silver. The spectra are vertically translated for easier visualization and appropriately labeled.

**Figure 5:**
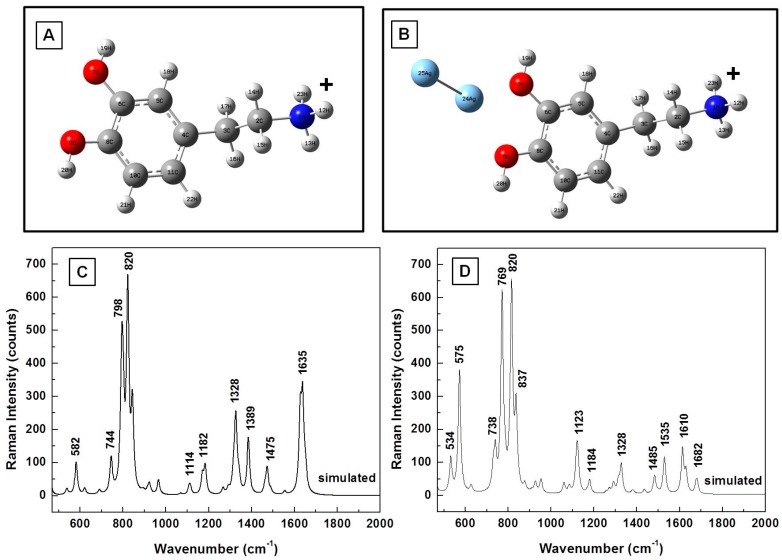
(**A**,**B**) Structural representations of the cationic form of dopamine without and with the silver dimer, respectively; (**C**,**D**) Theoretically calculated Raman vibrations of DA^+^ alone and in the proximity of silver, respectively.

In view of the fact that other SERS vibrational lines have been experimentally observed and reported in the literature for dopamine, and the dissociation of the DA^0^ molecule into DA^−^ and DA^+^ in water is also expected during sample preparation, the analysis of these ionic DA forms alone and in the presence of silver is presented in Figure 4A–D and Figure 5A–D. The energetically optimized structural representations of these anionic and cationic forms are shown in Figure 4A,B and in Figure 5A,B, respectively. Slight deformations (torsional and bending) of the amine chains are evident in both cases. Furthermore, while the silver dimer has a planar orientation in regard to the benzene ring and is asymmetrically closer to the oxygen in the anionic case, a quasi-planar position of the silver towards the primary ammonium NH_3_^+^ functional group is seen for the cationic form.

The simulated Raman vibrational frequencies in the regions of interest for DA^−^ and DA^+^, which are presented in Figure 4C and Figure 5C, respectively, show dominant peaks around 640, 750, 780, 930, 1150, 1280, 1530, and 1610 cm^−1^ for the anionic form and around 580, 800, 820, 1180, 1330, 1390, 1480, and 1640 cm^−1^ for the cationic form. The silver influence in the computed Raman vibrational lines, which is shown in Figure 4D and Figure 5D, reveals a decrease in the intensities of DA characteristic lines around 750 cm^−1^ and 780 cm^−1^ in the anionic case. The opposite behavior is observed for DA^+^, resembling more that of DA^0^. Small variations in the positions and intensities of other Raman lines are also seen in these figures. For a comparative analysis, an experimental Raman spectrum is also presented in Figure 4D. The spectra in this figure are again vertically translated for easier visualization and appropriately labeled.

An interesting observation concerns the computed line around 1150 cm^−1^, which has been previously measured [19,21]. The current results seem to indicate that it originates from in-plane bending of the anionic form of dopamine. This assumption better supports the notion of its presence in aqueous solution; however, during the drying process of the current sample preparation, some water molecules might remain attached to dopamine ions, and therefore not evaporate. As such, they could provide screening, hindering the re-association and neutralization of the charges. This screening could be quite strong, as it is proportional to the high dielectric constant of water (i.e., ε = 80). Another potential explanation is the creation of image charges in close proximity to the metallic Ag NPs, which can decrease the Born energy to a lower value than that of the entropy related to the dissociation of charges. As such, when the value of the entropy is large, the DA molecules remain charged and a dynamic equilibrium between the cationic and anionic forms of the dopamine molecules remains. Thermal energy acquired due to laser excitation could also be a contributing factor. Although we would not eliminate this possibility, we consider it less likely, as the laser power of 100 μW remained constant for all measurements.

**Figure 6:**
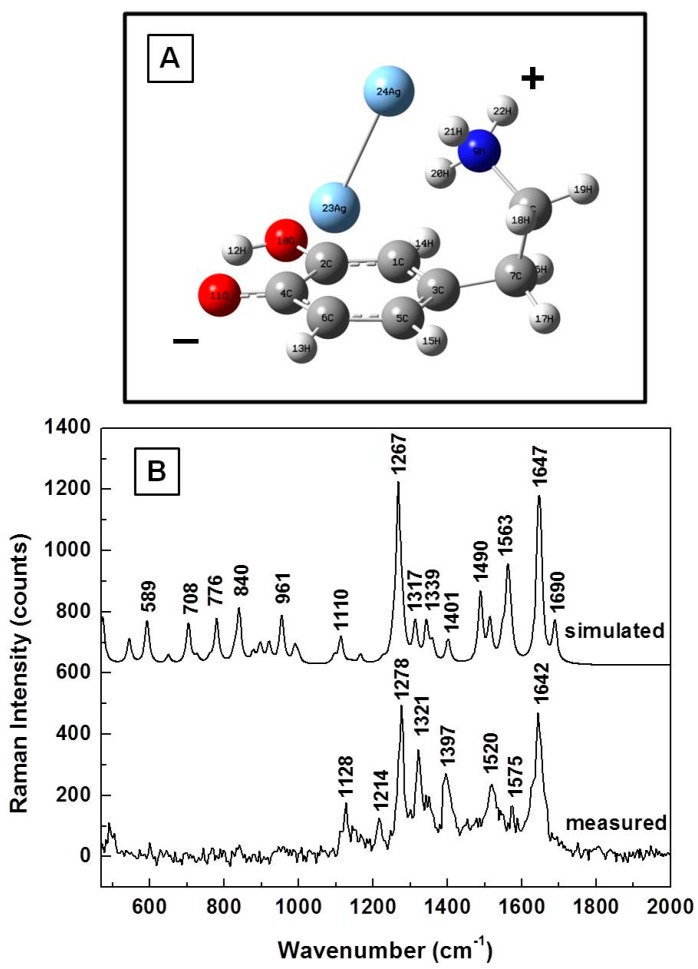
(**A**) Dopamine DA^±^ structural representation in the proximity of silver; (**B**) Theoretically calculated and experimentally measured Raman vibrations of DA**^±^**, as labeled. The spectra are vertically translated for easier visualization.

Charge redistribution and formation of intramolecular hydrogen bonds is also possible. Such an uncharged DA^±^ structure in the vicinity of the silver dimer is presented in Figure 6A, and is computationally and experimentally analyzed in Figure 6B. An obvious bending of the amine chain towards the silver dimer is observed in Figure 6A, with the dimer oriented quasi-perpendicularly to the benzene ring of the molecule. More notably, this energetically stable configuration suggests that dopamine could interact with the silver surface through both functional groups: the known proton donor hydroxyl group and the NH_3_^+^. Besides the SERS cases of dopaminequinonic and anionic forms, where no DA characteristic vibrations around 750 cm^−1^ and 790 cm^−1^ or their very slight presence were computationally obtained and experimentally confirmed, only their weak contributions are theoretically predicted in Figure 6B for this DA^±^ configuration.

The relatively good agreement between the calculated and measured results, which are also presented in Figure 6B, not only confirms the existence of this configuration, but also provides additional insights into DA morphological changes that might occur during analyte detection at physiological levels by other techniques such as electrochemical fast-scan cyclic voltammetry [1,21,22]. The current results suggest that while dopaminequinone is more likely detected electrochemically as the analyte’s oxidized form, when the potential is applied in the negative direction, the DA^±^ configuration is more probably detected as the reduced form. Furthermore, it is obvious that the analyte undergoes reorientation and deformation when adsorbed onto the silver surface, from a quasi-planar, vertically oriented structure with the oxygen atoms as direct adsorption sites (see dopaminequinone form in Figure 3B) to a quite contorted, bent structure with oxygen and nitrogen as adsorption sites (see DA^±^ form in Figure 6A).

**Figure 7:**
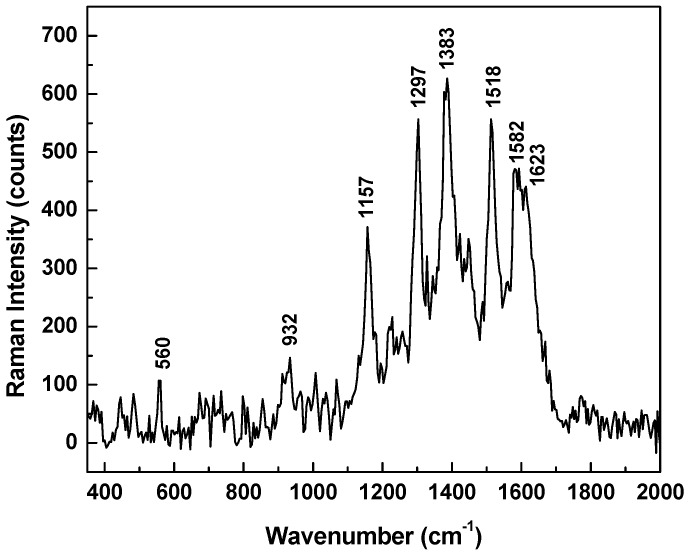
Overall average of 400 SERS spectra recorded in different spots on the sample (eight different time series acquisitions of 50 SERS spectra each, at 200 milliseconds per spectrum).

Characteristic signatures corresponding to each DA configuration can also be detected, such as the vibration lines around 950 and 1360 cm^−1^ for dopaminequinine, around 1160, 1290, and 1530 cm^−1^ for the anionic form, and around 1280 and 1640 cm^−1^ for DA^±^. In order to eliminate the random fluctuations expected in any experimental data, and for better comprehension of the likelihood of occurrence for these configurations, we present in Figure 7 the overall average of 400 SERS spectra, which were collected in different spots on the sample (eight different time series acquisitions of 50 SERS spectra each, at 200 milliseconds per spectrum). Considering the intensities of the vibrational lines observed in this spectrum, the dopaminequinone form appears to be the predominant configuration, followed by that of the anionic form, and that of the DA^±^ form; the latter two forms might occur with approximately the same proportions. However, due to the similarity in some of their signatures, such as the 1290 cm^−1^ line for the anionic form and the 1280 cm^−1^ vibration for the DA^±^ form, together with the typical variation in full width at half maximum (FWHM) of the recorded Raman features, it is difficult to determine a reliable numerical estimate of their contributions. In the context of this study, it is also worth mentioning that none of the characteristic neutral DA^0^ (standard DA powder, see Figure 1) signatures around 750 and 790 cm^−1^ were detected experimentally, as observed in this spectrum. Their occurrence would imply the potential presence of multilayer neutral DA^0^, or of cationic DA^+^.

## 4. Conclusions

To conclude, in this SERS study we present a comparative theoretical and experimental approach to the detection of dopamine at a concentration of 10^−11^ molar, which is definitely lower than normal physiological levels. Although the current measurements were not performed in aqueous solution, they facilitate a reliable understanding of vibrational assignments. Not only is the usual broadening of Raman bands that is characteristic of measurements in liquids eliminated in this case, but since the consideration of aqueous media would further complicate computational analysis, we have simplified the comparison and assessment of results. An even greater experimental challenge would be the question of obtaining a common consensus on the reproducibility of Raman intensities in a liquid environment. Especially at low concentrations, this issue involves the probability that molecules will become localized in regions of intense optical fields trapped between adjacent plasmonic surfaces, and thereby be subjected to SERS enhancement of their inherently weak Raman signals. By eliminating such limitations, we were able to acquire important insights into the complexity of dopamine detection at a very low concentration.

The relatively good agreement between the simulated and experimentally determined results suggests the possibility that different forms of the DA molecule, such as uncharged DA^±^, anionic DA^−^, as well as dopaminequinone, are present. All of these molecular configurations, in a SERS environment, also lose the dominant DA Raman lines at 750 cm^−1^ and 790 cm^−1^, suggesting their adsorption onto the metallic surface under different orientations. The occurrence of these features would imply the potential presence of multilayer neutral DA^0^, or that of cationic DA^+^. From a redox reaction perspective, which is known to take place in electrochemical measurements, the dopaminequinone form is more likely detected during the oxidation process, while the DA^±^ form more probably occurrs during reduction. The ultrasensitivity of the current experimental data, in combination with the theoretical analysis presented in this work, definitely provides valuable information for the advancement of the detection and monitoring of dopamine. 

## Figures and Tables

**Table 1:** Theoretically calculated and experimentally measured Raman vibrations of dopamine with their tentative assignments.

Calculated (cm^−1^)	Measured (cm^−1^)	Assignment
381	393	CH wagging, ring deformation
577	593	CH in-plane ring deformation
622	635	CH wagging; aliphatic chain C–C vibrations
744	750	CH out-of-plane; ring deformation (two band response)
782	795	CH out-of-plane; ring deformation (two band response)
934	931	NH twisting
961	962	NH twisting; CH wagging; ring deformation
1029		CH wagging
1060		C–C–N stretching; CH wagging
1123	1117	CH twisting; NH twisting; CN stretching
1156	1148	OH rocking; CH aromatic rocking; weak ring breathing; CH wagging
	1209	CO stretching
1294	1285	Ring breathing; CH aromatic rocking; CH twisting
1321		Ring breathing; CH aromatic in-plane rocking; CH twisting
1355		Ring deformation; OH scissoring; CH twisting
1387		CH wagging; NH twisting
1466	1455	CH scissoring
1615	1617	Ring deformation, OH scissoring
1631		NH_2_ scissoring
1634		Benzene ring deformation
